# Synaptic NMDA receptor activity is coupled to the transcriptional control of the glutathione system

**DOI:** 10.1038/ncomms7761

**Published:** 2015-04-09

**Authors:** Paul S. Baxter, Karen F.S. Bell, Philip Hasel, Angela M. Kaindl, Michael Fricker, Derek Thomson, Sean P. Cregan, Thomas H. Gillingwater, Giles E. Hardingham

**Affiliations:** 1 Centre for Integrative Physiology, University of Edinburgh School of Biomedical Sciences, Hugh Robson Building, George Square, Edinburgh EH8 9XD, UK; 2 Institute of Cell Biology and Neurobiology, Charité—Universitätsmedizin Berlin, Charitéplatz 1, 10117 Berlin, Germany; 3 Department of Pediatric Neurology, Charité—Universitätsmedizin Berlin, Augustenburger Platz 1, 13353 Berlin, Germany; 4 Department of Biochemistry, University of Cambridge, Tennis Court Road, Cambridge CB2 1QW, UK; 5 Department of Physiology & Pharmacology, The University of Western Ontario, J Allyn Taylor Centre for Cell Biology, 100 Perth Drive, London, Ontario, Canada N6A 5K8

## Abstract

How the brain’s antioxidant defenses adapt to changing demand is incompletely understood. Here we show that synaptic activity is coupled, via the NMDA receptor (NMDAR), to control of the glutathione antioxidant system. This tunes antioxidant capacity to reflect the elevated needs of an active neuron, guards against future increased demand and maintains redox balance in the brain. This control is mediated via a programme of gene expression changes that boosts the synthesis, recycling and utilization of glutathione, facilitating ROS detoxification and preventing *Puma*-dependent neuronal apoptosis. Of particular importance to the developing brain is the direct NMDAR-dependent transcriptional control of glutathione biosynthesis, disruption of which can lead to degeneration. Notably, these activity-dependent cell-autonomous mechanisms were found to cooperate with non-cell-autonomous Nrf2-driven support from astrocytes to maintain neuronal GSH levels in the face of oxidative insults. Thus, developmental NMDAR hypofunction and glutathione system deficits, separately implicated in several neurodevelopmental disorders, are mechanistically linked.

## Abstract (web summary)

How the brain’s antioxidant defenses adapt to changing demand is not well understood. Here the authors demonstrate that synaptic activity is coupled to transcriptional control of the glutathione antioxidant system via NMDA receptors, enabling neurons to tune their antioxidant defenses.

## Graphical abstract

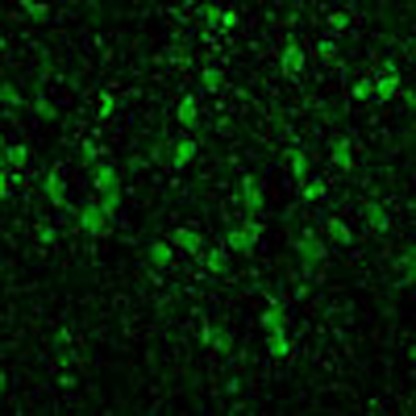

Glutathione plays a central role in maintaining cellular redox balance, both by reacting non-enzymatically with a variety of reactive oxygen species (ROS), and by acting as a cofactor in the glutathione peroxidase-catalysed reduction of peroxides^1^,^2^. Deregulation of glutathione homeostasis has been implicated in the aetiology of many brain disorders. Blood sample and post-mortem studies have revealed deficits in GSH in patients with a variety of neurodegenerative diseases^3^. Deficits in the GSH system have also been implicated in the pathophysiology of neuropsychiatric disorders, including schizophrenia (SZ), bipolar disorder (BD) and autistic spectrum disorder (ASD). Studies have reported that SZ, BD and ASD patients exhibit reduced GSH levels and various markers of oxidative stress^4^,^5^,^6^,^7^. Moreover, both genetic and pharmacological mouse models of GSH deficiency induce behavioural and cognitive disturbances relevant to these neurodevelopmental disorders^5^,^8^. Genetic evidence points to a potential role for GSH system deficits, particularly in SZ where disease-associated variants have been found in the *GCLC* gene, which encodes the catalytic subunit of glutamate–cysteine ligase (GCL) that catalyses the rate-determining step in GSH biosynthesis^9^.

It is thought that one of the harmful consequences of GSH system dysfunction in the brain is NMDA receptor (NMDAR) hypoactivity^4^. GSH enhances NMDAR responses whereas its depletion or oxidation, results in NMDAR hypofunction^10^,^11^. NMDAR hypoactivity is particularly deleterious during forebrain development, triggering vulnerability to neurodegeneration and long-lasting behavioural disturbances. This is relevant to neuropsychiatric disorders, since disruption to the glutamatergic synapse, and NMDAR hypofunction in particular, has been implicated in the aetiology of BD, ASD^12^,^13^ and, especially SZ^14^,^15^. Moreover, the NMDAR-antagonistic properties of ethanol may contribute to its acute neurotoxicity in models of fetal alcohol syndrome^16^.

Despite the importance of GSH in brain (patho)physiology, regulation of the GSH system is incompletely understood. Leaving aside the feedback inhibition of GCL by GSH, the wider issue of whether the capacity of the system to synthesize, utilize and recycle GSH is subject to dynamic control in the brain is less clear. Such regulatory mechanisms may be important for normal redox homeostasis in the brain, for example, to tune the capacity of the GSH system to the needs of the cell, or to guard against future demand. Here we show that such a mechanism exists in neurons. Highly active neurons have an increased demand for GSH, which is met by a coordinated programme of transcriptional changes that serve to enhance the capacity of the GSH system, mediated by Ca^2+^ influx through the NMDAR. NMDAR hypoactivity is found to promote GSH depletion and neurodegeneration in the developing brain due to a loss of NMDAR-dependent transcriptional support of the GSH biosynthetic capacity. Thus, developmental NMDAR hypofunction and glutathione system deficits, separately implicated in several neurodevelopmental disorders, are mechanistically linked.

## Results

### Neuronal GSH represses Puma-dependent apoptosis

**Figure 1:**
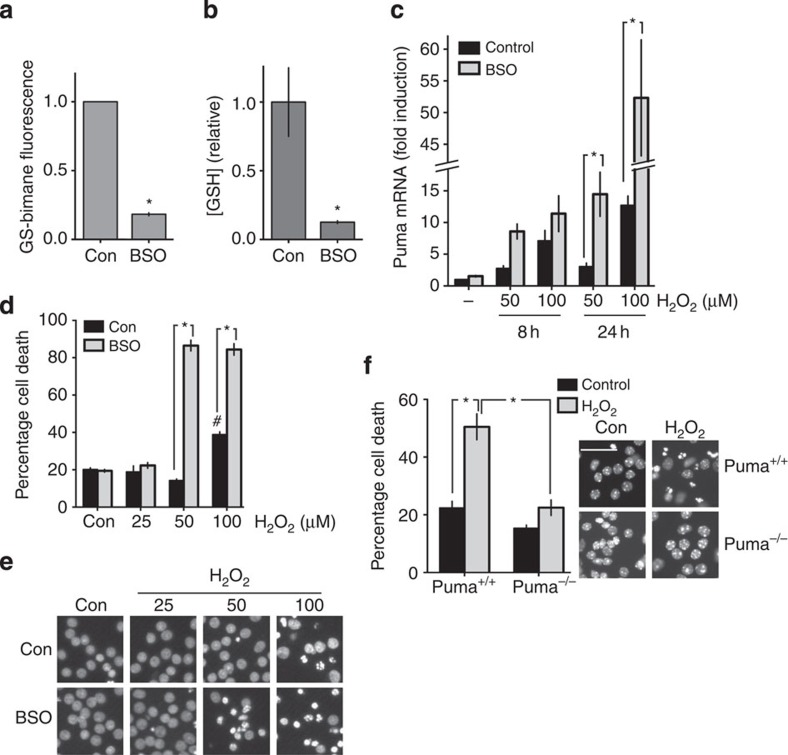
The GSH system is a major defense against Puma-dependent oxidative stress-induced apoptosis in developing cortical neurons. (**a**,**b**) Inhibition of GCL activity with BSO treatment depletes cortical neurons of glutathione. Cortical neurons were treated with BSO (200 μM here and throughout) for 24 h, after which GSH levels were measured using *in vivo* labelling with MCB or a colourimetric assay of total glutathione in cell-free extracts (see methods). *P=0.0009 (**a**, *n*=5), 0.013 (**b**) *n*=4), Student’s *t*-test here and throughout unless otherwise stated. Exact *P* values are quoted throughout unless *P*<0.0001. Mean±s.e.m. is shown here and throughout. (**c**) Oxidative stress-induced *Puma* mRNA expression is potentiated by GSH depletion. Neurons were treated with BSO for 24 h, then subsequently treated with 50 or 100 μM H_2_O_2_ and *Puma* expression analysed by qRT–PCR, normalized to *Gapdh*. *P=0.024, 0.005 referring to asterisks as shown from left to right (here and throughout). One-way analysis of variance followed by Fisher’s *post hoc* test (1WA-Fph), *n*=4. (**d**) Neuronal vulnerability to H_2_O_2_-induced death is potentiated by GSH depletion. Neurons were treated with BSO for 24 h, then treated with different concentrations of H_2_O_2_, with cell death analysed 24 h later. *P<0.0001, <0.0001, 1WA-FPh; #*P*=0.0352 compared with control condition (*n*=4 (BSO), *n*=2 (Con)). (**e**) Example pictures from **d**. (**f**) H_2_O_2_-induced neuronal apoptosis is Puma dependent. Puma^+/+^ and Puma^−/−^ cortical neurons were exposed to 100 μM H_2_O_2_. *P<0.0001, <0.0001; 2WA-FPh (*n*=6 Puma^+/+^; *n*=4 Puma^−/−^).

To investigate a link between synaptic activity and the GSH system, we first confirmed the extent to which the GSH system was central to antioxidant defenses in cortical neurons. We induced a GSH deficit by treating neurons overnight with buthione sulfoxamine (BSO), a selective inhibitor of GCL, the rate-determining enzyme of the GSH biosynthetic pathway. GSH levels were measured in cell extracts using a commercial colorimetric assay kit, and also the widely used cell-based probe monochlorobimane (MCB), a normally non-fluorescent dye which forms a (GST-catalysed) fluorescent adduct (GS-bimane) with GSH that can be measured fluorometrically^17^,^18^ (see Supplementary Fig. 1a–e, for validation of the MCB assay conditions). BSO treatment resulted in a reduction in GSH levels measured using either assay (Fig. 1a,b).

To determine the impact of GSH depletion on antioxidant defenses, we measured the vulnerability of cortical neurons to oxidative stress-induced apoptosis triggered by H_2_O_2_, a process that involves transcriptional induction of the BH3-only domain gene *Puma*^19^. We found that, while BSO-induced GSH depletion had no influence on basal levels of neuronal apoptosis or *Puma* expression, it strongly enhanced H_2_O_2_-induced Puma induction and apoptosis (Fig. 1c–e). *Puma* induction and apoptosis were observed in GSH-depleted neurons in response to low, ordinarily non-toxic doses of H_2_O_2_. The effects of BSO were rescued by treatment with a cell-permeable form of GSH (GSH-EE, Supplementary Fig. 1f). The effects were also similar to, and occluded by short interfering RNA-mediated *Gclc* knockdown (Supplementary Fig. 1g,h), confirming that the effects of BSO were due to decreasing GSH. We also confirmed that *Puma*-deficient neurons were resistant to H_2_O_2_-induced apoptosis, (Fig. 1f)^19^, and that *Puma*-deficient neurons had no GSH deficits (Supplementary Fig. 1i), ruling out any confounding effects of Puma deficiency on GSH antioxidant defenses. Collectively these data confirm the importance of the GSH system for cortical neuronal antioxidant defenses.

### Active neurons have a greater requirement for GSH

Synaptic activity is energetically and metabolically expensive^20^. Since ROS generation is a by-product of ATP production via oxidative phosphorylation, we hypothesized that the energy demands of sustaining strong synaptic activity that lead to increased ROS production cause elevated utilization of GSH. To investigate this, synaptic activity was enhanced by disinhibiting the cortical cultures by treatment with a GABA_A_ receptor blocker, bicuculline (BiC), which induces action potential bursting and concomitant intracellular Ca^2+^ transients dependent on NMDAR activity and augmented by release from internal stores^21^,^22^. Network disinhibition via BiC treatment results in phasic, transient elevation of glutamate, as illustrated by concurrent imaging of BiC-induced Ca^2+^ levels in neurons expressing GCAMP2 plated onto astrocytes expressing iGluSnFR^23^, an extracellular glutamate sensor. GCaMP2 and iGluSnFR spikes were found to be concurrent and transient (Supplementary Fig. 2b). In addition to BiC, the weak K^+^ channel blocker 4-aminopyridine (4-AP) was added to enhance burst frequency^21^.

**Figure 2:**
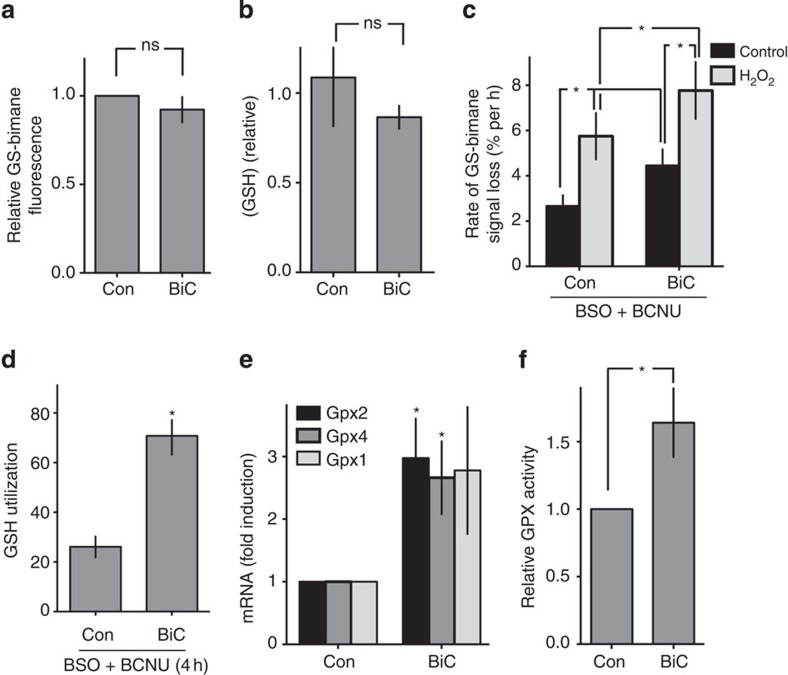
Electrically active neurons utilize more GSH and upregulate GSH peroxidase activity. (**a**,**b**) Synaptic activity does not alter steady-state basal GSH levels in unstressed neurons. Cortical neurons were stimulated with BiC/4-AP for 24 h, after which GSH levels were measured using *in vivo* MCB labelling (**a**) or a colourometric assay in cell-free extracts (**b**) see Methods, *n*=4 for both. (**c**) GSH utilization is enhanced by synaptic activity. Neurons were treated ±BiC/4-AP (BiC) for 24 h. Subsequently, cells were treated with BSO+BCNU (±H_2_O_2_) for 1, 2, 4, 8 or 12 h or left untreated. Thirty minutes before the end of this period of time, cells were loaded with MCB and GS-bimane fluorescence measured (normalized to protein). For each condition, GS-bimane fluorescence was plotted against time and the rate of decline in fluorescence (a measure of rate of GSH utilization) obtained by fitting a line to the data. *P=0.0039, 0.033, 0.021, 0.0029, 2WA-FPh (*n*=7). n.b., where one asterisk indicates two comparisons, the *P* value for the comparison closest to the asterisk is shown first. See Supplementary Fig. 2c and 2d for schematic of the experimental protocol and example of an experimental replicate used to obtain the rate of decline in GS-bimane fluorescence. (**d**) GSH utilization is enhanced by synaptic activity. Neurons were treated ±BiC/4-AP for 24 h. Subsequently, the cells were treated with BSO+BCNU for 4 h, after which GSH levels were determined using the colorimetric method and normalized to protein levels. *P=0.0058, (*n*=3). (**e**) Synaptic activity upregulates glutathione peroxidase 2 and 4 mRNA expression. Neurons were treated±BiC/4-AP for 24 h and expression of the indicated Gpx genes analysed. *P=0.017, 0.022 (*n*=10 Gpx2, *n*=5 Gpx4, *n*=9 Gpx1). (**f**) Glutathione peroxidase enzyme activity is increased by synaptic activity. Neurons were treated±BiC/4-AP for 24 h and GPX enzyme activity measured. *P=0.041 (*n*=8).

Neuronal electrical activity induces ROS production attributed to both cytoplasmic and mitochondrial sources^24^,^25^. Consistent with this, BiC/4-AP-induced synaptic activity enhanced rates of production of ROS (Supplementary Fig. 2a). We next studied the impact of synaptic activity on GSH levels and usage. Enhanced BiC-induced synaptic activity did not affect steady-state GSH levels even over a 24-h time period, measured either using an MCB assay or a colorimetric extract-based assay (Fig. 2a,b). However, these levels reflect the net effect of utilization, minus the rates of GSH biosynthesis and recycling (reduction). To gain a measure of GSH utilization, levels were measured under conditions where GSH biosynthesis and reduction were acutely inhibited, by BSO and 1,3-bis( chloroethyl)-1-nitrosourea (BCNU), respectively (see Supplementary Fig. 2c for a protocol schematic). In the presence of BSO and BCNU, the rate of GSH utilization, as assayed by the decline in GS-bimane signal, was higher in active neurons than in less-active ones (Fig. 2c, Supplementary Fig. 2d) indicating a greater rate of GSH utilization.

To determine whether active neurons were operating nearer to their maximum rate of GSH utilization, further demands on the system were imposed by exposing neurons to an oxidative insult (H_2_O_2_). We found that active neurons were able to respond to an H_2_O_2_ insult with still greater rates of GSH utilization, assayed by the decline in GSH-bimane signal (Fig. 2c) or by the decline in GSH levels by conventional extract-based assay (all in the presence of BSO and BCNU, Fig. 2d). Since a substantial amount of basal and H_2_O_2_-induced GSH utilization will be due to GSH peroxidation, we wanted to know whether neurons’ capacities for GSH peroxidation were increased by synaptic activity. We found that Gpx2 and Gpx4 were transcriptionally induced by synaptic activity (Fig. 2e) and, moreover, cell-free extracts taken from active neurons revealed higher levels of glutathione peroxidase activity than extracts from control cultures, including astrocyte-free cultures (Fig. 2f, Supplementary Fig. S2e). Thus, BiC-induced synaptic activity enhances GPX activity and increases both basal and peroxide-induced GSH utilization.

### Active neurons use more GSH

**Figure 3:**
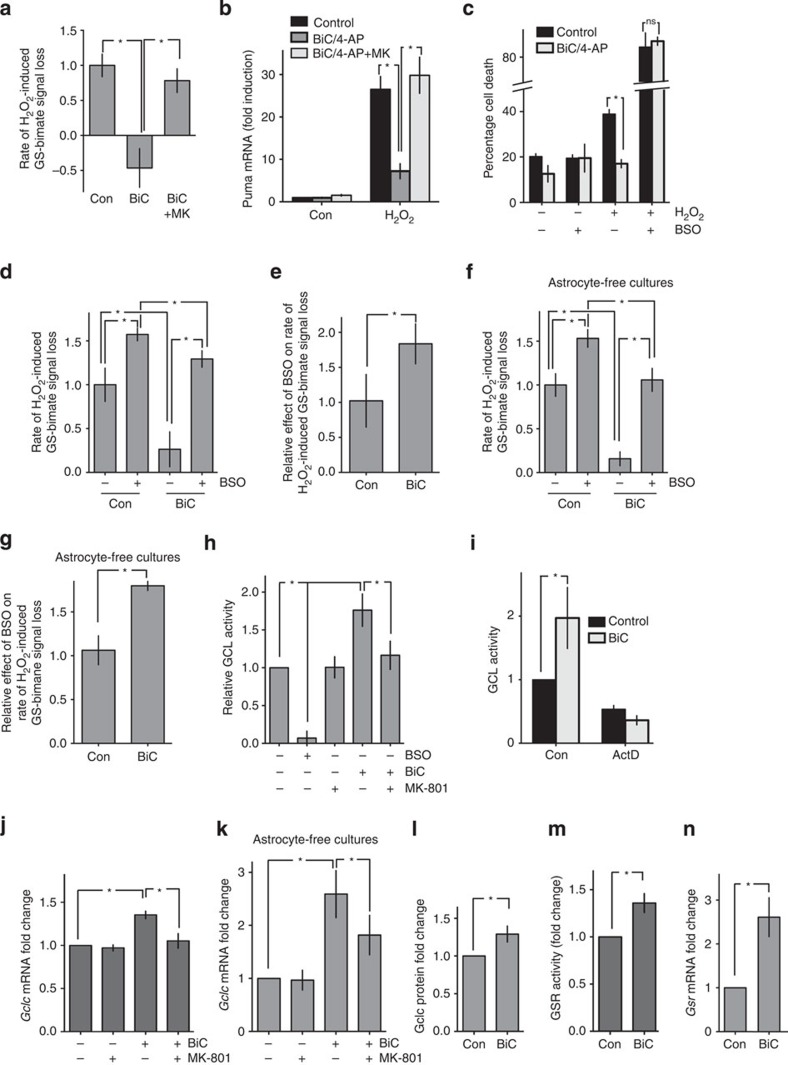
Synaptic activity boosts GSH biosynthesis and recycling. (**a**) NMDAR blockade inhibits the maintenance of GSH levels by synaptic activity. Neurons were treated for 24 h as indicated and the rate of decline in GS-bimane fluorescence induced by 100 μM H_2_O_2_ measured as described in Fig 2d. *P=0.0012, 0.0026, 1WA-Fph (*n*=7). (**b**) Synaptic activity inhibits oxidative stress-induced Puma mRNA expression in an NMDAR-dependent manner. Neurons were treated as indicated for 24 h, then with 100 μM H_2_O_2_ for 8 h (*P<0.0001, <0.0001, *n*=3 (BiC+MK-801), *n*=7 (BiC)). (**c**) Inhibition of glutathione synthesis blocks activity-dependent neuroprotection. Neurons were treated ±BiC/4-AP for 24 h±BSO; then with 100 μM H_2_O_2_ for 24 h. *P=0.037, 2WA-Fph (*n*=4 (BSO), *n*=2 (Con)). (**d**) Maintenance of GSH levels in active neurons is inhibited by GCL inhibition. Neurons were treated±BiC/4-AP, then rate of decline in GS-bimane fluorescence induced by 100 μM H_2_O_2_ measured as described in Fig. 2d ± BSO (added 30 min before H_2_O_2_ treatment). *P<0.0001, 0.0002, 0.0083, <0.0001, 2WA-Fph (*n*=7). (**e**) Using data from **d**, the effect of BSO on the rate of H_2_O_2_-induced GSH loss was calculated. *P=0.0043 (*n*=7). (**f**,**g**) As per **d**,**e** but performed on astrocyte-free neuronal cultures. *P=0.0021, 0.008, 0.011, 0.0017 (F), 2WA-Fph, *P*=0.017 (*n*=4). (**h**) Synaptic activity increases NMDAR-dependent GCL enzyme activity (24 h treatment). *P=0.026, 0.035, 0.047, 1WA-Fph (*n*=10 (Con, BiC), *n*=6 (MK, BiC+MK)). (**i**) Synaptic activity induced GCL enzyme activity increase requires *de novo* mRNA transcription. BiC/4-AP-induced GCL activity measured ±Actinomycin D. *P=0.021, 1WA-Fph (*n*=4). (**j**) Synaptic activity increases *Gclc* mRNA expression via NMDAR activity, normalized to *Gapdh*. *P=0.0012, 0.0032, 1WA-Fph (*n*=3). (**k**) As per **j**, except that astrocyte-free cortical neuronal cultures were used. *P=0.023, 0.045 (*n*=5). (**l**) Synaptic activity induces Gclc protein expression in astrocyte-free cortical neuronal cultures, normalized to beta-actin. *P=0.022 (*n*=9). (**m**) Synaptic activity (24 h) increases glutathione reductase enzyme activity. *P=0.011 (*n*=9). (**n**) Synaptic activity increases glutathione reductase mRNA expression. *P=0.036 (*n*=4).

While the increased peroxidation of GSH is important in neutralizing harmful peroxide species, this could theoretically lead to faster depletion of GSH. We studied H_2_O_2_-induced GSH depletion in control neurons and neurons that had experienced 24 h of BiC-induced elevated activity. Remarkably, we found that while in control neurons GSH depletion was substantial, active neurons displayed no GSH depletion (Fig. 3a). Thus, despite active neurons generating more ROS and using more GSH basally and more rapidly in response to oxidative insults (Fig. 2), they are better able to maintain GSH levels in the face of increased demand (Fig. 3a). The effects of synaptic activity were inhibited by MK-801, placing the NMDAR as a key mediator of these adaptive changes (Fig. 3a). To further confirm the effects of synaptic activity on the intrinsic GSH system, we performed an experiment employing Grx1-roGFP2, which reports in real time the glutathione redox potential through its coupling to redox-sensitive GFP via its fusion to glutaredoxin^26^. This biosensor is highly sensitive, capable of detecting nanomolar changes in oxidized GSSG against a background of millimolar reduced GSH^26^. In neurons expressing Grx1-roGFP2, this high sensitivity leads to a saturating response on exposure to low concentrations of H_2_O_2_ (≥50 μM). However, by employing a lower dose (15 μM), we can monitor the response of the probe in control neurons compared with neurons that have experienced 24 h of BiC-induced burst activity. We found that the perturbation of the GSH redox potential induced by 15 μM H_2_O_2_ was significantly lower in the more active neurons than in the control neurons (Supplementary Fig. 3a–c). This supports the notion that the capacity of the GSH system is enhanced by synaptic activity.

We next studied the influence of synaptic activity on vulnerability to H_2_O_2_ insults in the presence or absence of chronic BSO pre-treatment (to deplete GSH levels). BiC-induced synaptic activity strongly repressed H_2_O_2_-induced *Puma* induction (Fig. 3b) and protected neurons against consequent apoptosis (Fig. 3c). However, in GSH-depleted neurons, activity-dependent protection was abolished (Fig. 3c), demonstrating the importance of the GSH system in activity-dependent protection against oxidative insults, likely in conjunction with alterations to other antioxidant systems^27^. In addition, we observed that NMDAR blockade abolished the effects of BiC-induced synaptic activity, both in terms of preventing H_2_O_2_-induced *Puma* induction (Fig. 3b), preventing H_2_O_2_-induced GSH depletion (Fig. 3a), and H_2_O_2_-induced neuronal death (Supplementary Fig. 3d). Note that the antagonist of the major group I mGluR in cortical neurons (mGluR5) MTEP did not prevent activity-dependent protection ((Supplementary Fig. 3d), while the AMPAR antagonist CNQX (which prevents BiC/4-AP-induced bursting that leads to NMDAR activation) did inhibit protection (Supplementary Fig. 3d). Collectively these data indicate that NMDAR synaptic activity boosts either GSH biosynthesis, or the recycling (reduction) of oxidized glutathione, or both. These changes clearly more than compensate for elevated GSH utilization due to synaptic activity (Fig. 2c,d), since they enable GSH levels to be maintained even in the face of exogenous oxidative insults.

### Activity induces GSH biosynthesis and recycling

To assess whether synaptic NMDAR activity boosts GSH biosynthesis, we first studied the impact of acutely blocking GSH biosynthesis (BSO treatment) just before application of the oxidative insult. Acute BSO treatment increased the rate of H_2_O_2_-induced GSH depletion in both highly active and less-active (control) neurons (Fig. 3d), consistent with ongoing biosynthesis being an important part of neuronal GSH homeostasis^28^. Significantly, however, the magnitude of the effect of blocking GSH biosynthesis was greater in active neurons than less-active neurons (Fig. 3e), indicating that the rates of GSH biosynthesis were greater in active neurons.

Since our standard cortical culture preparation contains a mixture of around 90–95% neurons and 5–10% astrocytes^27^,^29^, we wanted to confirm that the effects of synaptic activity were mediated by the neurons themselves. We repeated the experiment in Fig. 3d using neuronal cultures essentially devoid of astrocytes (achieved by addition of an antimitotic agent on the day of plating) and obtained qualitatively similar results (Fig. 3f). Moreover, the magnitude of the effect of blocking GSH biosynthesis by BSO treatment was greater in highly active neurons than less-active neurons (Fig. 3g), as was observed in mixed cultures, indicating that activity-dependent increases in GSH biosynthesis is intrinsic to the neurons themselves.

To directly analyse the activity of the GSH biosynthetic pathway, we assayed, in cell extracts, the activity of GCL (the rate-limiting step in GSH biosynthesis). Extracts taken from neurons experiencing strong synaptic activity in the previous 24 h exhibited substantially more GCL activity than did extracts from control neurons (Fig. 3h). This activity-dependent increase in GCL activity was blocked by the transcription inhibitor Actinomycin D (Fig. 3i), suggestive of a role for *de novo* gene expression. Indeed, synaptic NMDAR activity increased expression of *Gclc*. Consistent with a direct effect on the transcriptional activity of the *Gclc* promoter, activity of a luciferase-based reporter of the *Gclc* promoter *Gclc*-Luc^30^ was induced by BiC/4-AP stimulation (Supplementary Fig. 3f). We also wanted to study the regulation of *Gclc* mRNA in astrocyte-free neurons. Because astrocytes produce more GSH than neurons and express more GCL^1^, we wanted to determine whether the presence of astrocytes was partly masking the true extent of activity-dependent induction of Gclc in neurons. *Gclc* mRNA levels in astrocyte-free neuronal cultures were found to be around half of that in mixed cultures (5–10% astrocytes) and seven times lower than those in pure astrocyte cultures (Supplementary Fig. 3g). Importantly, BiC-induced synaptic activity triggered an increase in *Gclc* mRNA and protein expression in astrocyte-free cultures (Fig. 3k,l, Supplementary Fig. 3e). Collectively, these data indicate that activity-dependent induction of neuronal GSH biosynthetic capacity plays a key role in sustaining GSH levels in the face of increased demand.

While enhanced biosynthesis induced by synaptic activity clearly plays a major role in maintaining GSH levels in the face of increased utilization, we also considered whether synaptic activity boosts GSH recycling. We noted that GSH utilization following H_2_O_2_ exposure in the presence of BSO was still lower in active neurons than control (Fig. 3d), whereas in the presence of both BSO and BCNU the reverse was true (Fig. 2c). This indicates that synaptic activity is also boosting GSH recycling, a process whereby oxidized GSSG is reduced to GSH in a reaction catalysed by glutathione reductase. Indeed, BiC-induced synaptic activity was found to increase GR enzyme activity in neuronal cultures in the presence (Fig. 3m) or absence of astrocytes (Supplementary Fig. 3h), induce *Gsr* mRNA expression (Fig. 3n) and also induce activity of a luciferase reporter of the Gsr promoter (Supplementary Fig. 3i).

### Cooperation between activity and astrocytic Nrf2

Forebrain neurons receive significant antioxidant support from surrounding astrocytes^31^. This non-cell-autonomous support is regulated by a gene expression programme controlled by the transcription factor NF-E2-related factor 2 (Nrf2 (refs 31, 32). Activation of Nrf2-mediated gene expression in astrocytes, either via overexpression, pharmacological activation or activation by stressors such as oxidative stress or brief ischaemia confers non-cell-autonomous protection to surrounding neurons in a variety of models, including rodent models of neurodegenerative disease, as well as human stem cell-derived neuron-astrocyte systems^29^,^31^,^33^. The mechanism of protection involves the increased synthesis of astrocytic GSH, which is released, broken down and taken up by neurons to be used as precursors for their own GSH synthesis^34^. As such, this non-cell-autonomous support still requires neurons to have robust intrinsic systems for the synthesis of GSH. Indeed, astrocytic protection of neurons is abrogated by neuronal Gclc knockdown^35^. This raises the possibility that astrocytic Nrf2 activation (in boosting astrocytic GSH synthesis and supply of GSH precursors to neurons), would cooperate with synaptic activity (in boosting neuronal GSH biosynthetic capacity) in enabling the maintenance of neuronal GSH levels in the face of oxidative insults.

**Figure 4:**
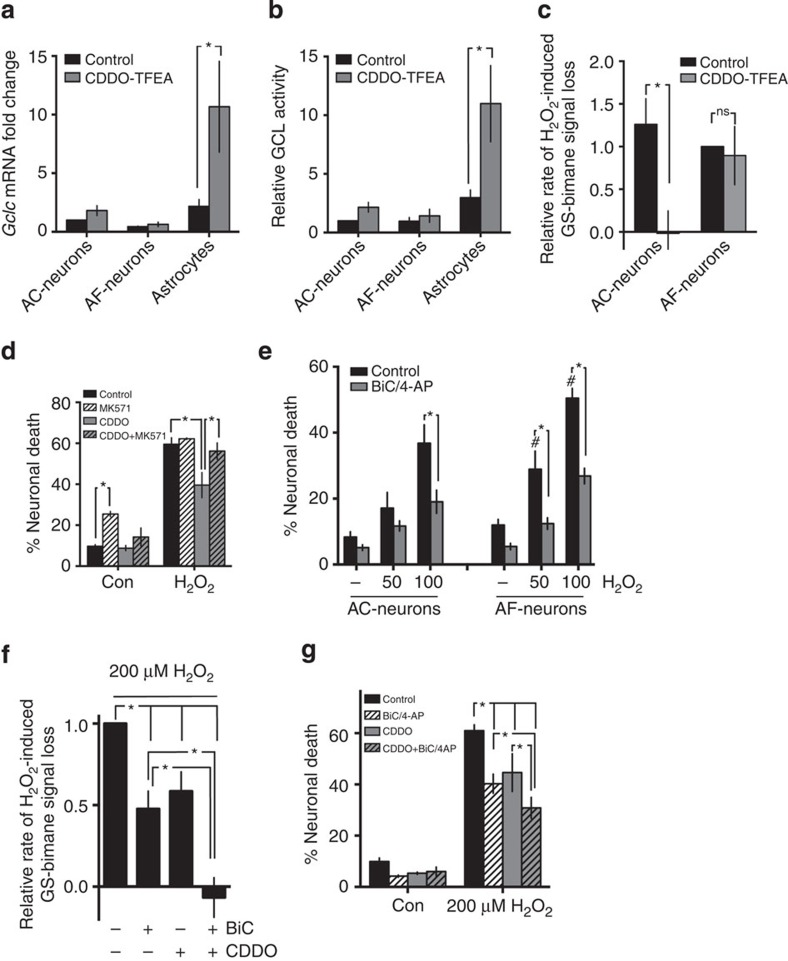
Cooperation between synaptic activity and astrocytic Nrf2 activators in supporting neuronal GSH levels. (**a**,**b**) The Nrf2 activator CDDO^TFEA^ induces Gclc expression and GCL activity in astrocytes but not in neurons. Regular astrocyte-containing (AC) cultures (5–10% astrocytes), astrocyte-free (AF) neuronal cultures and astrocyte cultures were treated with CDDO^TFEA^ (250 nM) for 24 h after which *Gclc* mRNA (*P*=0.0033, 2WA-Fph, *n*=4) and GCL activity (*P*=0.0002, 2WA, Fph, *n*=5) were assayed. (**c**) CDDO^TFEA^ protects neuronal cultures against H_2_O_2_-induced GSH loss via an astrocyte-dependent mechanism. AC- and AF-neuronal cultures were treated ±CDDO^TFEA^ for 24 h after which the rate of GSH loss induced by 100 μM H_2_O_2_ was measured by MCB assay. *P=0.0274, 2WA-Fph (*n*=5). (**d**) Neurons were treated ±CDDO^TFEA^±MRP1 inhibitor (MK571, 10 μM) and H_2_O_2_ (100 μM)-induced neuronal death induced 24 h later. *P=0.0027, 0.003, 0.0017, 2WA-Fph (*n*=4). (**e**) Astrocyte-free neurons still exhibit activity-dependent protection against oxidative stress but display elevated overall vulnerability. AC- and AF-neuronal cultures were treated ±BiC/4-AP for 24 after which the indicated concentrations of H_2_O_2_ were applied and cell death analysed 24 h later. *P<0.0001 for all; ^#^*P*=0.0007, 0.0002 comparing AF-neuronal death with equivalent AC-neuronal death level, 1WA-Fph (*n*=6). (**f**) Synaptic activity and Nrf2 activation by CDDO^TFEA^ cooperate to prevent GSH depletion in astrocyte-containing neuronal cultures. Regular astrocyte-containing neuronal cultures were treated ±BiC/4-AP±CDDO^TFEA^ as indicated for 24 h after which the rate of GSH loss induced by 200 μM H_2_O_2_ was measured by MCB assay. *P=0.0005, 0.0207, <0.0001, 0.0003, 0.0006, 1WA-Fph (*n*=4–12). (**g**) Synaptic activity and Nrf2 activation by CDDO^TFEA^ cooperate to prevent oxidative stress-induced death in astrocyte-containing neuronal cultures. Regular astrocyte-containing neuronal cultures were treated ±BiC/4-AP±CDDO^TFEA^ as indicated for 24 h after which 200 μM H_2_O_2_ was applied and cell death analysed 24 h later. *P=<0.0001, <0.0001, <0.0001, 0.0096, 0.0003, 2WA-Fph (*n*=8).

To investigate this, we used a potent Nrf2 activator, the triterpenoid 1[2-Cyano-3,12-dioxool-eana-1,9(11)-dien-28-oyl] trifluoroethylamide (CDDO^TFEA^ (ref. 36)). We recently showed that CDDOTFEA protects cortical neurons against H_2_O_2_-induced neuronal death in a Nrf2-dependent manner^37^. Moreover, the neuroprotective actions of CDDO^TFEA^ were found to be mediated by astrocytes^37^. Consistent with this, CDDO^TFEA^ treatment boosts Gclc mRNA expression and GCL activity in rat astrocytes but not in neurons (Fig. 4a,b). Moreover, CDDO^TFEA^ treatment prevented H_2_O_2_-induced GSH depletion in rat neuronal cultures containing astrocytes but not in astrocyte-free neuronal cultures (Fig. 4c). In addition, we found that astrocyte-mediated CDDO^TFEA^-induced protection is inhibited by an inhibitor of MRP1 (MK571), responsible for astrocytic GSH efflux^1^ (Fig. 4d). This is consistent with the known GSH-dependent mechanism of astrocytic Nrf2-mediated neuroprotection^34^,^37^.

The astrocyte dependency of the effects of CDDO^TFEA^ in preventing H_2_O_2_-induced GSH depletion contrasts strongly with the astrocyte-independent actions of synaptic activity in preventing GSH depletion and inducing Gclc expression (Fig. 3f,l). Consistent with this, we observe activity-dependent neuroprotection against H_2_O_2_-induced neuronal death in astrocyte-free cultures (Fig. 4e). We noted though that in astrocyte-free neuronal cultures, overall vulnerability is increased, compared to astrocyte-containing cultures (Fig. 4e). This both confirms that astrocyte support can be valuable and strongly suggests that astrocytic support and activity-dependent protection may be additive. We therefore investigated the effect of combining the cell-autonomous effect of BiC/4-AP-induced synaptic activity with the non-cell-autonomous, astrocyte-dependent action of CDDO^TFEA^. We used a strong insult (200 μM H_2_O_2_) designed to expose any cooperativity. Importantly, we found that while both synaptic activity and CDDO^TFEA^ separately promoted neuroprotection and reduced GSH depletion, combined their effect was greater than either treatment alone (Fig. 4f,g). To conclude, these data support the concept that synaptic activity-dependent induction of the intrinsic neuronal antioxidant defenses can act in concert with support provided by nearby astrocytes.

### *In vivo* NMDAR blockade represses GSH biosynthesis

**Figure 5:**
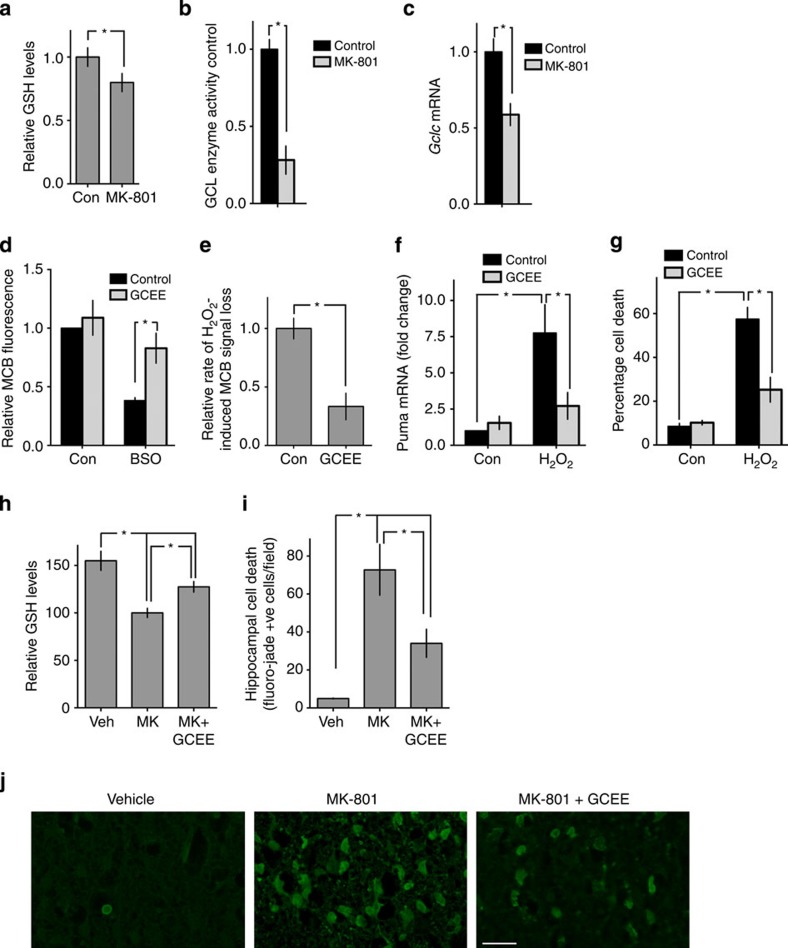
Deleterious effects of NMDAR blockade *in vivo* are due to *Gclc* transcriptional repression. (**a**) Blockade of NMDARs causes a reduction of cortical GSH content *in vivo*. Cortical GSH levels measured in P6 rat pups 24 h after the first injection. *P=0.0495, one-tailed *t*-test (*n*=4). (**b**,**c**) Blockade of NMDARs reduces GCL enzyme activity and *Gclc* expression *in vivo*. Rat pups treated as in Fig. 4a and cortical GCL activity (**b**) and Gclc expression (**c**) were measured at 12 h and expressed relative to the mean of the control group. *P*=0.0006 (**b**), 0.0042 (**c**) (*n*=4). (**d**) GCEE sustains GSH levels in the presence of GCL inhibitor. Neurons were treated with GCEE (1 mM) for 1 h, followed by 200 μM BSO for 24 h, followed by MCB assay. *P=0.0002 (*n*=9 (con), *n*=5 (BSO)). (**e**) GCEE attenuates GSH depletion by oxidative insult. Neurons were treated for 1 h with GCEE then rate of decline in GS-bimane fluorescence induced by 250 μM H_2_O_2_ measured. *P=0.0070 (*n*=4). (**f**,**g**) GCEE attenuates oxidative stress-induced Puma mRNA expression and cell death. Neurons were preincubated ±GCEE for 1 h, then treated with 100 μM H_2_O_2_ and either *Puma* levels. *P=0.0086, 0.047 (**f**) *n*=9 (con), *n*=5 (GCEE)) or cell death analysed.*P=0.0110, 0.0217 (*n*=4 (H_2_O_2_, *n*=3 (Con)). (**h**) GCEE rescues NMDAR blockade dependent forebrain GSH depletion *in vivo*. P6 Rat pups were injected twice at 0 and 8 h and cortical GSH levels measured at 24 h, normalized to protein content and expressed relative to MK-801 treated samples. *P=<0.0001, 0.0105, 0.0087, 1WA-Fph (*n*=8,9,9 for Veh, MK, MK+GCEE respectively). (**i**,**j**) GCEE rescues neurons from MK-801 induced cell death *in vivo*. P6 Rat pups were injected twice at 0 and 8 h and killed 24 h post first injection, followed by Fluoro-Jade staining-based analysis of neurodegeneration within the hippocampus. *P=<0.0001, 0.0105, 0.0087, 1WA-Fph (*n*=9, 11, 11 for Veh, MK and MK+GCEE, respectively). Scale bar, 30 μm.

We next investigated the extent to which synaptic NMDAR activity regulates the GSH system *in vivo* in the developing brain. We studied the effects of inducing NMDAR hypoactivity by administrating the NMDAR antagonist MK-801 to P7 rats. MK-801 administration led to a reduction in total glutathione levels in the forebrain (Fig. 5a). Moreover, analysis of the activity of GSH pathway enzymes revealed that NMDAR hypoactivity led to a strong reduction in GCL activity in the brain (Fig. 5b), which was also associated with a strong reduction in *Gclc* mRNA expression (Fig. 5c). Expression of *Gsr* mRNA was also repressed by MK-801 administration (Supplementary Fig. 4a) but GR enzyme activity was not repressed (Supplementary Fig. 4b), potentially due to changes in mRNA not yet reflected at the protein level. Collectively these data suggest that NMDAR hypoactivity leads to a deficit in the GSH biosynthetic pathway in developing neurons due to a requirement for NMDAR activity to support expression of Gclc. Administration of NMDAR antagonists to rodents within the first two postnatal weeks has been consistently shown to induces an increase in apoptosis in certain brain regions, including the hippocampus^38^. Our observations regarding the role of the NMDAR in coupling synaptic activity to activation of the GSH biosynthetic pathway, via transcriptional induction of *Gclc* raised the possibility that dysregulation of GSH biosynthesis underlies some of the deleterious effects of NMDAR hypoactivity in the developing brain. We next tested this hypothesis.

### Rescue of degeneration triggered by *in vivo* MK-801

Since GCL catalyses the production of γ-glutamyl cysteine (GCEE), we reasoned that we may be able to bypass any deficits in GCL activity by supplying the brain with a cell-permeable (monoethyl ester) form of γ-GCEE. Before performing *in vivo* experiments, we assessed the efficacy of GCEE supplementation on cultured cortical neurons, finding that it inhibited the depletion of GSH levels in neurons treated with the GCL inhibitor BSO (Fig. 5d). Thus, supplying neurons with γ-GCEE does indeed bypass neurons’ requirement for GCL. Moreover, GCEE supplementation also substantially reduced the rate of H_2_O_2_-induced GSH depletion in control neurons (Fig. 5e), and prevented *Puma* induction (Fig. 5f), essentially mimicking the effect of synaptic activity. As expected, GCEE was also neuroprotective against H_2_O_2_-induced apoptosis (Fig. 5g). As a caveat, it is important to note that γ-GCEE can act as an antioxidant directly as well as providing the final precursor for GSH synthesis^39^.

We next investigated the effect of GCEE supplementation on the effects of NMDAR hypoactivity *in vivo*, focusing on the observed reduction in GSH levels and on the acute increase in neuronal apoptosis observed on administration of MK-801 at doses similar to those that also cause long-lasting neurobehavioral deficits^38^,^40^,^41^. We found that co-administration of GCEE with MK-801 partly reversed the loss of GSH induced by NMDAR hypoactivity (Fig. 5h), demonstrating functional activity of GCEE in the brain, consistent with previous studies^42^. We then investigated whether inhibiting the NMDAR hypoactivity-induced GSH deficit had any effect on the acute increases in apoptosis known to be triggered at this developmental stage. Focusing on the hippocampus, we observed a marked increase in neurodegeneration 24 h after MK-801 administration (Fig. 5i,j), consistent with previous studies^38^. Importantly, levels of apoptosis were lowered by around 50% by administration of GCEE (Fig. 5i,j). Thus, while we cannot rule out the effects of GCEE in non-neuronal cells, these rescue experiments show that in the developing brain, the deficits in GSH biosynthesis induced by NMDAR hypoactivity are causally linked to neuronal loss.

## Discussion

In this study, we have shown that synaptic NMDAR activity plays a key role in regulating the capacity of the glutathione-based antioxidant system in developing neurons. Synaptic activity is energetically expensive, placing an ATP demand on the neuron, which must be met by increased metabolic activity, particularly oxidative phosphorylation^20^. In development, these demands are particularly strong, since synaptic activity may also be coupled to extensive structural changes such as neurite outgrowth/aborization, synaptogenesis and experience-dependent plasticity. Neuronal electrical activity is known to induce ROS production^24^,^25^, which explains why highly active neurons exhibit higher rates of GSH utilization (Fig. 2c,d).

These observations provide a plausible biological explanation for why the GSH system should be subject to activity-dependent control. If ROS generation in active neurons is higher, then it makes sense for the capacity of neurons to synthesize, recycle and utilize GSH to also be increased to ensure the correct redox balance in the neuron. The increased capacity produced is greater than the increased demand, since synaptic activity protects neurons against additional exogenous insults, potentially representing an added safeguard against unexpected increased demand. Given the deleterious effects of GSH depletion, the transcriptional control of GSH system components described likely represent a major part of neurons’ adaption of their antioxidant defenses, in concert with changes to the thioredoxin–peroxiredoxin system^27^,^43^,^44^. The increased resilience provided by enhanced antioxidant defenses may be augmented by the activity-dependent regulation of pro- and anti-apoptotic genes^45^,^46^, although of note, we observe activity-dependent protection against both apoptotic and non-apoptotic ROS-induced death (induced by low and high ROS respectively).

The role of the NMDAR activity in boosting GSH biosynthesis adds to our knowledge regarding the neuroprotective actions of physiological synaptic NMDAR activity, which contrast with the deleterious effects of chronic NMDAR activation (particularly extrasynaptic)^45^,^47^. One can speculate that clinically well-tolerated NMDAR antagonists such as memantine, which favour the antagonism of harmful chronic NMDAR activity over phasic activation^48^,^49^ would interfere with the GSH system less than conventional antagonists. Memantine is used to treat Alzheimer’s disease and shows promise in preclinical models of Huntington’s disease^48^,^50^. Since both are diseases associated with GSH deficits^3^, it would be important to not interfere with signals that support GSH biosynthesis.

NMDAR hypoactivity (induced by a variety of antagonists) during development triggers both acute pathological and long-lasting behavioural disturbances in rodents, such as an acute increase in apoptotic-like neuronal death in a variety of brain regions^38^ as well as long-term behavioural and cognitive effects extending into adulthood, including deficits in prepulse inhibition, increased perseverative behaviour and cognitive dysfunction. While the NMDAR-antagonistic properties of ethanol contribute to its acute neurotoxicity in models of fetal alcohol spectrum disorders^16^, milder NMDAR hypofunction during development has emerged as a prominent hypothesis for the aetiology of SZ, partly as a result of the above behavioural deficits being relevant to SZ, as well as the earlier observations that NMDAR antagonists transiently reproduced key psychomimetic and behavioural symptoms in humans^14^. Furthermore, a reduction in cortical parvalbumin-positive interneurons, a hallmark of SZ, can also be triggered by NMDAR antagonists^14^, suggesting that they may be particularly vulnerable to NMDAR hypofunction (the relatively young age of the rats used in the current study prevented analysis of parvalbumin-positive interneurons since they are yet to develop). Indeed, such enhanced vulnerability to early-life oxidative stress has been demonstrated^51^. It is possible that deficits in parvalbumin-positive interneurons may arise from milder episodes of NMDAR hypofunction than those required to cause substantial neuronal death. More recently, genetic evidence, particularly genome-wide association study, has implicated the NMDAR subunit gene *GRIN2B* and regulators of NMDAR activity (such as the Neuregulin-ErbB pathway) as susceptibility genes for SZ^14^. Furthermore, molecular studies into the function of SZ risk gene *DISC1* (ref. 52) indicate a potential role in controlling NMDAR function, such as via its interaction with serine racemase (which generates the NMDAR co-agonist D-serine^53^) or regulation of PDE4B–PKA–CREB pathway-dependent GluN2A expression^54^.

The mechanism by which NMDAR hypoactivity results in neuronal loss and dysfunction in the developing brain is incompletely understood. Unlike in the adult, where necrotic neuronal death induced by NMDAR hypoactivity is likely due to excitotoxic disinhibition of GABAergic transmission, apoptotic neuronal death in the developing brain (with immature GABAergic circuits) is likely due to a cell-autonomous requirement for synaptic NMDAR activity. Our current study supports a model whereby transcriptional deregulation of GSH biosynthesis contributes partly to the deleterious effects of NMDAR hypoactivity, potentially in concert with transcriptional repression of neuroprotective erythropoietin^38^. It is also not clear what determines the developmental window of vulnerability of the developing brain to NMDAR hypoactivity (circa P4-P21)^38^. This window coincides broadly with the early part of developmental synaptogenesis during which circuits are formed, strengthened and refined. Synaptic activity is energetically expensive^20^ requiring high rates of metabolism and involving high levels of Ca^2+^ influx, risking elevated ROS generation that must be detoxified. Our study shows that, consistent with this, the rates of GSH utilization are indeed higher in highly active neurons, making adaptive changes to the supply side of the GSH system key to redox homeostasis. Another possibility is that the decline in vulnerability to NMDAR blockade in later development reflects additional antioxidant support supplied by astrocytes, which are still proliferating during this period. Indeed, astrocyte-mediated antioxidant support mediated by Nrf2-dependent antioxidant gene expression is a major contributor to neuronal resistance to oxidative insults in the mature brain^31^. Moreover, our study here illustrates that both regulation of intrinsic defenses within neurons, in combination with astrocytic-derived support, is optimal for providing resilience to oxidative insults.

Our findings that the NMDAR couples synaptic activity to GCL-dependent GSH biosynthesis in developing neurons is particularly interesting given that GSH deficits and GCL variants are already implicated in the pathophysiology of SZ^5^. GSH levels are lower in the cerebrospinal fluid of SZ patients and also in the prefrontal cortex (PFC)^4^,^5^, and recent magnetic resonance spectroscopy studies found that lowered GSH levels were associated with stronger negative symptoms among SZ patients^5^. Recently, a correlation between GSH levels in the PFC and white matter integrity along the cingulum bundle was demonstrated in both control and early psychosis patients^55^, while a correlation between GSH levels and resting-state functional connectivity was only observed in control subjects. This is significant given the known association of SZ with myelin deficits and loss of connectivity in the PFC, and can be attributed at the molecular level to a GSH deficit-induced impairment of oligodendrocyte progenitor proliferation and maturation^55^.

Genetic evidence also supports a role for GSH system deficits in SZ. Fibroblasts from SZ patients show a deficit in the induction of *GCLC*^9^. Moreover, a significant association has been reported between SZ and a CAG trinucleotide repeat polymorphism in the *GCLC* 5′ untranslated region. Allele combinations that conferred a high risk of SZ were also associated with reduced induction of GCL activity, GCLC expression and GSH levels in patient-derived fibroblasts^9^. Of note, the product of GCL, γ-GCEE, can act in place of GSH in GPX1-catalysed peroxide reduction^39^, further underlining its utility as a key antioxidant. The genetic associations with *GCLC* are particularly intriguing in the light of studies that show disease-relevant phenotypes in mouse models of GSH deficiency, triggered by both genetic and pharmacological means^5^. Mice deficient in Gclm have reduced levels of GSH, and display enhanced responses to a psychostimulant (amphetamine), altered stress responses and social behaviour, impaired prepulse inhibition and deficits in associative learning^5^,^8^. Moreover, both genetic and pharmacologically induced models of GSH deficiency exhibit impaired object recognition memory^5^.

The consequences of GSH deficits relevant to the pathophysiology of SZ include parvalbumin-positive interneuron dysfunction and deficits in myelination maturation, that can be reversed by juvenile antioxidant treatment^4^,^51^,^55^. Moreover, the relevance of redox imbalance to SZ is further strengthened by the recent demonstration that juvenile antioxidant treatment prevents electrophysiological and behavioural deficits in a developmental model of SZ (neonatal ventral hippocampal lesion^56^). Another effect of GSH deficits relevant to SZ pathophysiology is impairment of NMDAR activity^4^. The NMDAR contains redox-active cysteine residues, with currents potentiated by reducing conditions and inhibited by oxidizing agents, including oxidized glutathione GSSG^11^. GSH and its metabolites also have reported direct actions on the ionotropic glutamate receptors^57^. Moreover, depletion of GSH causes a selective reduction of NMDAR currents and impairment of synaptic plasticity^4^. It is becoming apparent that NMDAR hypoactivity and GSH deficits have a reciprocal relationship, with one positively feeding back onto the other. Transient NMDAR hypoactivity during development, which can reduce GSH levels^58^,^59^, may trigger redox imbalance and further repression of the NMDAR^4^. Downstream consequences of NMDAR hypoactivity and redox imbalance, such as alterations to the excitation–inhibition balance of certain forebrain circuits, could contribute to the pathological phenotype.

To conclude, the fact that the GSH system can be transcriptionally controlled by synaptic activity via the NMDAR means that the capacity of the system can easily adapt to the increased metabolic demands of active neurons. However, a consequence of this is that NMDAR hypoactivity during development leads to a deficit in this important antioxidant system.

## Methods

### Neuronal culture and synaptic activation

Cortical neurons from E21 Sprague–Dawley rats were cultured as described^60^,^61^ and experiments performed at 8–10 DIV. *Puma*-knockout neurons were prepared from E17 *Puma*-null founder mice obtained from Professor Andreas Strasser^62^. To obtain astrocyte-free cultures the antimitotic agent cytosine arabinoside (Sigma) was added to the cultures on the day of plating (DIV0) rather than the usual DIV4. This results in <0.2% GFAP-positive astrocytes rather than the usual 5–10% (ref. 29). Cortical astrocyte cultures were prepared as previously described^63^. Before stimulations, neurons were transferred to a trophically deprived medium (22 (Tmo) containing 10% MEM (Invitrogen) and 90% Salt/Glucose/Glycine medium consisting of: 114 mM NaCl, 0.219% NaHCO_3_, 5.292 KCl, 1 mM MgCl_2_, 2 mM CaCl_2_, 10 mM HEPES, 1 mM glycine, 30 mM glucose, 0.5 mM sodium pyruvate, 0.1% phenol red; osmolarity 325 mOsm l^−1^). Bursts of action potentials were induced through stimulation with 50 μM BiC and 250 μM 4-aminopyridine (BiC/4-AP), which in turn disinhibits the neuronal network and depolarizes the cells, generating high frequency action potential firing^22^. The following reagents were used: buthionine sulfoximine (BSO), carmustine (BCNU), MK-801 were purchased from Tocris, BiC and H_2_O_2_ from Sigma, γ-glutamylcysteine-ethyl ester (GCEE) from Bachem, 4-aminopyridine from Calbiochem. To quantify cell death, neurons were fixed and subjected to nuclear DAPI (Vectorlabs) staining, then imaged using a Leica AF6000 LX imaging system with a DFC350 FX digital camera. Cell death was quantified by counting (blind) the number of pyknotic nuclei as a percentage of the total, with ∼1,500 cells counted per treatment.

### *In vivo* MK-801 administration

All procedures were authorized under a UK Home Office approved project licence and adhered to regulations specified in the Animals (Scientific Procedures) Act (1986). Seven day-old (P7) Sprague–Dawley rats of both sexes received two intraperitoneal injections of saline vehicle, or 0.5 mg kg^−1^ MK-801 at 0 and 8 h, in addition to (where indicated) GCEE (75 mg kg^−1^). At 12 h or 24 h after the first injection, rats were killed and frontal cortices were either collected and snap-frozen in liquid nitrogen for processing for GSH content, enzyme assays or mRNA expression or fixed (3% paraformaldehyde, 4% sucrose in PBS) and paraffin imbedded for Fluoro-Jade staining, carried out as described previously on 10-μm coronal sections^64^. Note that previous studies have already found that MK-801 administration in the P7 rat brain involves the degeneration of neurons and not astrocytes^38^. A high level of widespread neurodegeneration was prominent in the hippocampus of MK-801 treated pups, particularly in the dorsal aspects of the CA1 region. Cell death within this anatomical region was quantified via manual (blind) counting of the total number of Fluoro-Jade C positive fluorescent neurons within at least two fields of interest per section. In each pup, at least three to five non-adjacent coronal sections were quantified for hippocampal Fluoro-Jade C positivity. The total Fluoro-Jade C+ cell counts for each quantified coronal section were then averaged into a single data point representing neurodegeneration in each animal (number of fluorescent cells per field area).

### Glutathione and GST assays

Cellular glutathione content was measured using two methods. A colorimetric assay kit was used (Cayman Chemical Company) following an established method described^65^. Post stimulation, the cells were lysed over ice in 70 μl assay buffer (0.2 M 2-(*N*-morpholino)-ethanesulphonic acid, 0.5 mM K_2_HPO_4_, 1 mM EDTA, pH 6) plus 0.5% Triton-X-100, then centrifuged at 15,700*g* at 4 °C for 10 min. For brain samples, tissue was defrosted and lysed on ice using a dounce homogenizer in 10 μl of lysis buffer per mg tissue. Samples were then mixed with an equal volume of metaphosphoric acid (10 g per 100 ml) and centrifuged at 580*g* (2,500 r.p.m.) at 4 °C for 5 min to precipitate protein; and 200 mM triethanolamine was added to supernatants. Samples were then transferred to a clear 96-well plate and the reaction was started by adding assay buffer with GSH reaction mixture (containing NADP+, glucose-6-phosphate, glucose-6-phosphate dehydrogenase, glutathione reductase and Ellman’s reagent). Production of 5-thio-2nitrobenzoic acid, (a yellow reaction product from reduction of Ellman’s reagent with GSH) was measured for 20 min with a FLUOstar OPTIMA (BMG Labtech, Aylesbury, UK). A portion of lysate, taken from before protein precipitation, was set aside to determine protein concentration.

Alternatively, a fluorescent cell-based assay using MCB (Sigma) was performed. MCB is a membrane-permeable dye that forms a fluorescent product when bound to thiols; however, in the presence of glutathione *S*-transferase, it specifically becomes conjugated to GSH (forming fluorescent GS-bimane) at a rate several orders of magnitude faster^18^,^66^. Thirty minutes before the end of stimulation, neurons were treated with 50 μM MCB, and allowed to incubate at 37 °C. Cells were then washed once with fresh TMo, and lysed in K_2_HPO_4_ buffer containing 0.5% Triton-X-100. Lysates were centrifuged at 15,700*g* (13,000 r.p.m.) at 4 °C for 10 min, and supernatants were transferred to a black 96-well plate for fluorescence measurement (excitation 405 nm, emission 520 nm) with a FLUOstar OPTIMA. Lysates were then assayed for protein concentration using a BCA assay, to which fluorescence values were normalized to.

The following experiments were performed to validate the MCB assay conditions as an accurate way of measuring intracellular GSH levels. We confirmed that the MCB concentration (50 μM, 30′) is not limiting: incubation of neurons with a cell-permeable ethyl ester form of GSH (GSH-EE) increases the cellular GS-bimane signal (Supplementary Fig. 1a, the effect of GSH depletion (24 h BSO treatment) is shown for contrast). We also compared the generation of GS-bimane fluorescence signal (50 μM, 30′) with an extended incubation time (50 μM, 60′) and an elevated MCB concentration (250 μM, 30′). We found that the GS-bimane fluorescence signal using these two alternative conditions was modestly higher-around 20% (50 μM, 60′) and 40% (250 μM, 30′) higher, respectively (Supplementary Fig. 1b). However, we found that accompanying this slightly higher signal was a greater proportion of signal remaining after GSH depletion by 24 h BSO treatment (Supplementary Fig. 1c). To determine the true degree of GSH depletion 24 h BSO treatment, we used an alternative GSH assay (Promega’s GSH-glo assay), which is a quantitative assay whose linear dose response was confirmed using a standard curve of serial GSH dilutions (as per manufacturer’s instructions). Using the GSH-glo method, we found that 24-h BSO treatment resulted in only 15±1% of GSH remaining compared to control (*n*=4). This figure was much closer to that observed with our original conditions (50 μM, 30′), with the remaining signal in the other two conditions (50 μM, 60′ and 250 μM, 30′) being rather higher. This disproportionate increase in the apparently nonspecific (GSH-independent) signal with longer incubation times and higher MCB concentrations led us to conclude that the 50 μM, 30′ conditions were near optimal. In addition, to show approximate linearity in response to the MCB assay, we performed an experiment where parallel sets of neurons were treated for different times (2, 6 and 24 h) with BSO with GSH levels measured by MCB assay and GSH-Glo assay in parallel. The close relationship between GSH levels and the GSH-Glo assay were confirmed in all experiments by a standard curve. Moreover, as can be seen below, there is a good correlation between the MCB assay and the parallel GSH-glo data (Supplementary Fig. 1e). For example, a 6-h incubation with BSO results in GSH depletion of 20–30%, as observed using both assays.

For live-cell imaging of the GSH redox potential, neurons expressing Grx1-roGFP2 were subject to live-cell imaging on a Leica AF6000 LX imaging system and DFC350 FX digital camera during treatment with H_2_O_2_. Pairs of images were taken (ex 387±5 and 494±10; em 530±10 in both cases) and the ratio calculated.

In addition, since formation of the GS-bimane adduct requires GST activity, we studied GST activity (GST Assay Kit, Cayman) and found no influence of BiC/4-AP-induced burst activity on GST activity (Supplementary Fig. 1d). This is consistent with our observation that GSH levels measured by the MCB assay (Con versus BiC/4-AP) were unchanged (Fig. 2b) as they were when using the ‘Ellman's Reagent’ GSH assay (Fig. 2c). Any change in GST activity would likely have resulted in a discrepancy between the two results.

### GCL assay

To determine GCL activity, neurons were treated as indicated for 24 h and the enzyme activity was recorded as described^67^. In brief, cell cultures were lysed over ice in 650 μl lysis buffer (20 mM Tris, 1 mM EDTA, 250 mM sucrose, 20 mM sodium borate, 2 mM L-serine (borate and serine added to inhibit γ-glutamyl transpeptidase activity^68^), then centrifuged at 15,700*g* (13,000 r.p.m.) at 4 °C for 10 min. For brain samples, tissue was defrosted and lysed on ice using a dounce homogenizer in 10 μl of lysis buffer per mg tissue. Supernatants were then redistributed to 50 μl aliquots, one for each time point, and placed at 37 °C in a heat block. For BSO samples, 15 μl of 100 mM BSO was added to 285 μl of control supernatant and thoroughly mixed by vortex before redistribution. Fifty μl of GCL reaction buffer (400 mM Tris, 40 mM L-glutamic acid, 2 mM EDTA, 20 mM sodium borate, 2 mM L-serine, 40 mM MgCl2, 40 mM ATP, pH 7.4) was added to each sample and allowed to incubate for 5 min. Reaction was started by adding 50 μl of 20 mM cysteine to samples and incubated for 20, 15, 10 and 5 min. The GCL reaction was arrested by adding 50 μl of metaphosphoric acid (2.5 g per 100 ml) to precipitate protein, samples were subsequently vortexed and placed on ice for 20 min. After incubation, samples were centrifuged at 580*g* (2,500 r.p.m.) at 4 °C for 5 min. Following centrifugation, 20 μl of reaction mixture was transferred to a black 96-well plate, and 180 μl of detection buffer (50 mM Tris (pH 10), 0.5 N NaOH and 10 mM 2,3-Napthalenedicarboxyaldehdye (Sigma) (v/v/v—1.4/0.2/0.2)) was added to each well. The 2,3-Napthalenedicarboxyaldehdye in this mixture rapidly forms a fluorescent cyclic reaction product with the cysteine thiol and glutamyl amino groups of GSH and GCγ (*λ*_excitation max_=472 nm, *λ*_emission max_=528 nm)^69^. The plate was left to incubate in the dark at room temperature for 30 min, and fluorescence intensity was measured (excitation 485 nm, emission 520 nm) with a FLUOstar OPTIMA (BMG Labtech, Aylesbury, UK). GCL activity was determined by calculating rate of fluorescence increase over time, normalized to protein content. Note that the BSO-treated samples were used as an initial negative control to confirm that the assay was indeed measuring GCL activity. BSO presence was found to inhibit the signal produced by the assay by 93±9% (*n*=4). Having confirmed this, BSO was not routinely used for subsequent experiments.

### Glutathione peroxidase assay

To determine glutathione peroxidase (GPX) activity, a kit was used (Calbiochem) following the method described^70^. Post stimulation, cells were lysed in 50 μl of lysis buffer (20 mM HEPES pH 7.9, 100 mM KCl, 300 mM NaCl, 10 mM EDTA, 0.1% Nonidet P-40) and centrifuged at 15,700*g* (16,400 r.p.m.) at 4 °C for 10 min. Subsequently, 20 μl of sample was distributed to a well of a clear 96-well plate, with 70 μl of assay buffer (50 mM Tris-HCl, pH 7.6, 5 mM EDTA), 50 μl of co-substrate mixture (NADPH, glutathione and glutathione reductase), with glutathione peroxidase as a positive control and lysis buffer as a negative control. Using a multichannel pipette 20 μl of cumene hydroperoxide was added to each well starting the reaction. NADPH absorbance was then read every 30 s for 15 min using FLUOstar OPTIMA (BMG Labtech, Aylesbury, UK). GPX activity was determined by calculating rate of NADPH loss over time, normalized to protein content.

### Glutathione reductase assay

To determine glutathione reductase activity NADPH oxidation was recorded as a loss of absorbance at 340 nm as described. Post stimulation cells were lysed in 50 μl of lysis buffer (20 mM HEPES pH 7.9, 100 mM KCl, 300 mM NaCl, 10 mM EDTA, 0.1% Nonidet P-40) and centrifuged at 15,700*g* (16,400 r.p.m.) at 4 °C for 10 min. Subsequently, 20 μl of sample was distributed to a well of a clear 96-well plate, with 20 μl of 1 mM GSSG and 110 μl of assay buffer (100 mM Potassium Phosphate, pH 7.0), with a 0.1-U ml^−1^ solution of yeast glutathione reductase (Sigma) as a positive control and lysis buffer as a negative control. Using a multichannel pipette 50 μl of 1 mM NADPH was added to each well starting the reaction. NADPH absorbance was then read every 30 s for 15 min using FLUOstar OPTIMA (BMG Labtech, Aylesbury, UK). GR activity was determined by calculating the rate of NADPH loss over time, normalized to protein content.

### MitoSOX imaging

Neurons were loaded with 3 μM MitoSOX (Invitrogen) for 15 min, followed by extensive washing. Note that since MitoSOX oxidation, and mitochondrial oxidative stress in general, can arise from ROS generated in the cytoplasm, MitoSOX oxidation is simply a metric of cellular ROS, and not of mitochondrial ROS in particular. Mitosox fluorescence images were taken using a Leica AF6000 imaging system with a DFC350 FX digital camera (ex/em 530/590 nm). The rate of fluorescence increase (MitoSOX oxidation), relative to initial fluorescence, was calculated before and after BiC/4-AP stimulation within 4 min windows.

### RNA isolation and quantitative RT-PCR

This was performed as described previously^27^. RNA was isolated using the Roche isolation reagents including a 15-min DNase I treatment to avoid genomic DNA contamination of samples, and eluted using 50 μl of RNase-free water. For quantitative PCR (qPCR), complementary DNA (cDNA) was synthesized from 1 to 5 μg of RNA with the Tanscriptor One-Step RT–PCR Kit (Roche). In brief, 7 μl of RNA was mixed on ice with 10 μl 2 × cDNA Synthesis master mix, random primers: oligo primers 2:1 (total 3 μl), 2 μl deoxynucleotide mix (1 mM each: dATP, dTTP, dCTP and dGTP), 0.5 μl RNase inhibitor (40 U μl^−1^), 0.5 μl reverse transcriptase (20 U μl^−1^) and 3 μl nuclease-free water. Reaction mixtures were vortexed and spun down and run in parallel with at least one ‘no RT’ control. Samples were placed in a thermal cycler and incubated for 10 min at 25 °C, 30 min at 55 °C, 5 min at 85 °C and were then cooled down to 4 °C. This cDNA was then diluted to 6 ng μl^−1^ for use in real-time quantitative PCR. qPCR was performed in an Mx3000P qPCR System (Stratagene) using 2 × FastStart Universal SYBR Green Master Mix (Roche) according to the manufacturer’s instructions. In brief, a total volume of 15 μl qPCR reaction mix was added per well, containing 1 μl template DNA, SYBR Green master mix (7.5 μl, containing ROX), 0.6 μl of forward and reverse primers at 200 nM final concentration and 5.3 μl nuclease-free water. Technical replicates, NoRT controls and template-free controls were included in each case. The cycling programme was 10 min at 95 °C; 40 cycles of 30 s at 95 °C, 40 s at 60 °C with detection of fluorescence and 30 s at 72 °C; followed by one cycle of 1 min at 95 °C, 30 s at 55 °C ramping up to 95 °C over 30 s with continuous fluorescence detection (for dissociation curve). Expression of the gene interest was calculated, normalizing to either GAPDH or 18 s as a housekeeping gene. Primers used are shown below.


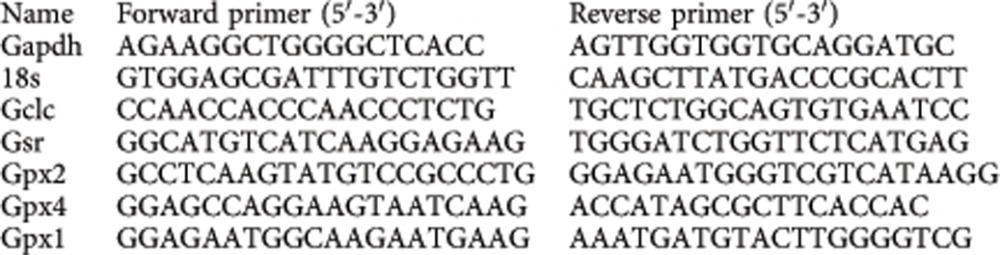


### Transfection and reporter assays

Neurons were transfected at DIV8 using Lipofectamine 2000 (Invitrogen) as described in Qiu, 2013 (ref. 60), using a total of 0.6 μg cDNA per well. For luciferase reporter assays, 0.1 μg of either pTK Renilla or p-sv40 Renilla were transfected along with 0.5 μg of a 1.1 kb Gclc promoter luciferase construct (a gift from Professor Naomi Fukagawa^30^, or mouse Gsr promoter luciferase construct, a gift from Professor Shyam Biswal. Luciferase assays were performed using the Dual Glo assay kit (Promega) with Firefly luciferase-based reporter gene activity normalized to Renilla control.

### Western blotting

Neurons were lysed in sample buffer (1.5 M Tris, 3% SDS, 15% glycerol, 7.5% β-mercaptoethanol, 0.0375% bromophenol blue, pH 6.8) and boiled for 4 min at 100 °C. Gel electrophoresis and western blotting was performed using an Xcell Surelock system with 4–20% NuPage BisTris pre-cast gels (Invitrogen). Post protein migration, the gels were blotted onto polyvinylidene difluoride membranes (Millipore) and then blocked for 1 h at room temperature in 5% (w/v) non-fat dried milk in TBS with 0.1% Tween 20. Membranes were then incubated overnight at 4 °C in blocking solution and diluted primary antibodies: Gclc (1:1000, Abcam), β-actin (1:2000, Abcam). Membranes were then washed in TBS with 0.1% Tween 20, and incubated with the appropriate horseradish peroxidase-conjugated secondary antibody for 1 h at room temperature. Membranes were washed again, and visualized by incubating in LumiGlo reagent and peroxide (Cell Signalling Technology) and exposing Kodak X-Omat film to the membranes.

### Statistical analysis and equipment settings

Statistical testing involved a two-tailed paired Student’s *t*-test. For studies employing multiple testing, we used analysis of variance followed by Fisher’s least significant difference *post hoc* test. For western blots, we used chemiluminescent detection on Kodak X-Omat film. Appropriate exposures were taken such that bands were not saturated. For figure preparation of example western blots, linear adjustment of brightness/contrast was applied (Photoshop) equally across the entire image, taking care to maintain some background intensity. Pictures of transfected neurons were taken on a Leica AF6000 LX imaging system, with a DFC350 FX digital camera. The DFC350 FX digital camera is a monochrome camera, and so coloured images (for example, of green fluorescent protein) essentially involve taking a black and white image (using the appropriate filter set) and applying a colour to the image after capture. All luminescent assays were performed on a FLUOstar OPTIMA (BMG Labtech, Aylesbury, UK). Light collection time and gain were set such that counts were substantially lower than the maximum level collectable. All chemicals were obtained from Sigma Aldrich (Gillingham, UK) unless otherwise stated.

## Additional information

**How to cite this article:** Baxter, P. S. *et al.* Synaptic NMDA receptor activity is coupled to the transcriptional control of the glutathione system. *Nat. Commun.* 6:6761 doi: 10.1038/ncomms7761 (2015).

## Supplementary Material

Supplementary Information Supplementary Figures 1-4
