# Corrigendum: Synaptic NMDA receptor activity is coupled to the transcriptional control of the glutathione system

**DOI:** 10.1038/ncomms16158

**Published:** 2017-09-11

**Authors:** Paul S. Baxter, Karen F. S. Bell, Philip Hasel, Angela M. Kaindl, Michael Fricker, Derek. Thomson, Sean P. Cregan, Thomas H. Gillingwater, Giles E. Hardingham

Nature Communications 6: Article number: 6761 ; DOI: 10.1038/ncomms7761 (2015); Published 04 09 2015; Updated 09 11 2017.

Michel Goedert, who developed the Thy1-P301S transgenic mouse, was inadvertently omitted from the Acknowledgments section of this Article. The Acknowledgements should have included the following:

‘We thank Michel Goedert for providing the Thy1-P301S transgenic mouse that was used in this study.’

