# Supplementary material for: Synaptic NMDA receptor activity is coupled to the transcriptional control of the glutathione system
Source: Nat Commun. 2015 Apr 9;6:6761. doi: 10.1038/ncomms7761 (PMC4403319; doi:10.1038/ncomms7761)
Supplement: Supplementary Information — Supplementary Figures 1-4 [file ncomms7761-s1.pdf]

Supplementary Figures

Fig. S1

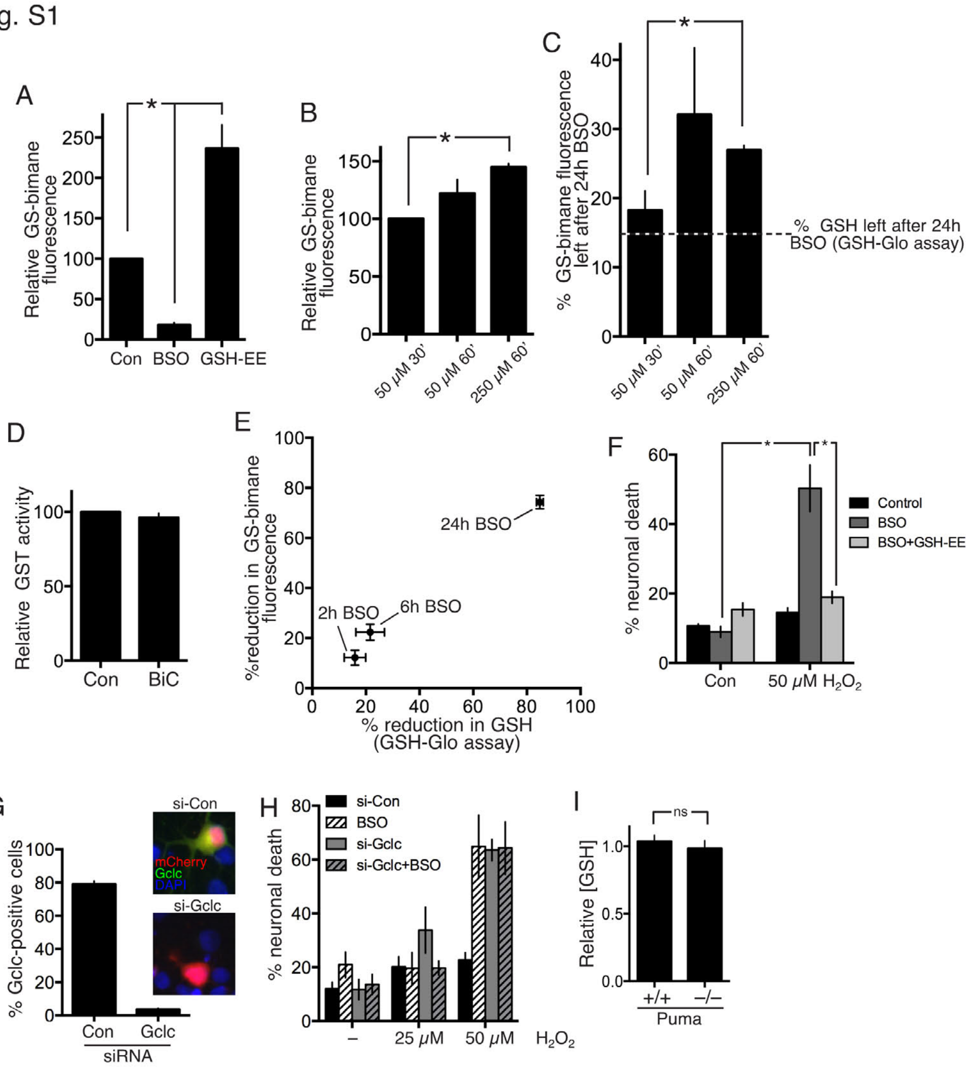

Supplementary Figure 1, relating to Figure 1.

A) Neurons were treated with BSO for 24h or GSH-EE (2 mM, 4h) and an MCB assay performed (50 µM MCB, 30'). *p<0.0001, 0.043 (n=3-5). **B)** An MCB assay was performed on parallel cultures with the indicated MCB concentrations and loading times. *p=0.0008, 1WA-FPh (n=4-5). **C)** Neurons were treated ± BSO (24h) and an MCB assay performed with the indicated assay conditions (i.e. different MCB concentrations and loading times). The % fluorescence remaining after BSO treatment was calculated relative to the control for that particular assay ocndition. The dotted line indicates the level of GSH remaining after 24h BSO incubation, as measured by GSH-Glo assay (Promega), an assay which was validated by a GSH standard curve *p=0.0008, 1WA-FPh (n=4-5). **D)** Neurons were treated ± BiC/4-AP (24h) were lysed and a GST assay performed (n=3). **E)** Parallel cultures were treated with BSO for the indicated times and subject to MCB assay and GSH-Glo assay respectively. The % signal remaining after BSO treatment was calculated (n=5). **F)** Neurons were treated where indicated with BSO ± GSH-EE (24h) then subsequently treated with 50 µM H_2O_2 and cell death analysed after a further 24h. *p=0.0009, 0.0024 (n=3). **G)** Neurons were transfected with 100 nM Control or 100 nM SMARTpool: siGENOME Rat Gclc siRNA (Thermo Fisher) plus plasmids encoding mCherry and myc-tagged Gclc. After 48h, the neurons were processed for immunofluorescence with an anti-Myc antibody and the efficacy of the Gclc siRNA measured by calculating the % of transfected (mCherry-positive) cells that also expressed myc-Gclc (n=6). Inset shows example images. **H)** Neurons were transfected with Con or Gclc siRNA, plus a eGFP marker. At 24h they were treated ± BSO and at 48h pictured taken prior to H_2O_2 treatment. Pictures were taken after a further 24h and the neurons scored as live/dead as described [Martel, 2012 #3016]. For each condition 120-280 cells were analysed across n=4 experiments. **I)** GSH levels in Puma$^{-/-}$ cortical neuronal extracts were measured by GSH-Glo assay and expressed relative to the levels in cultures made from Puma$^{+/+}$ littermates.

Fig. S2

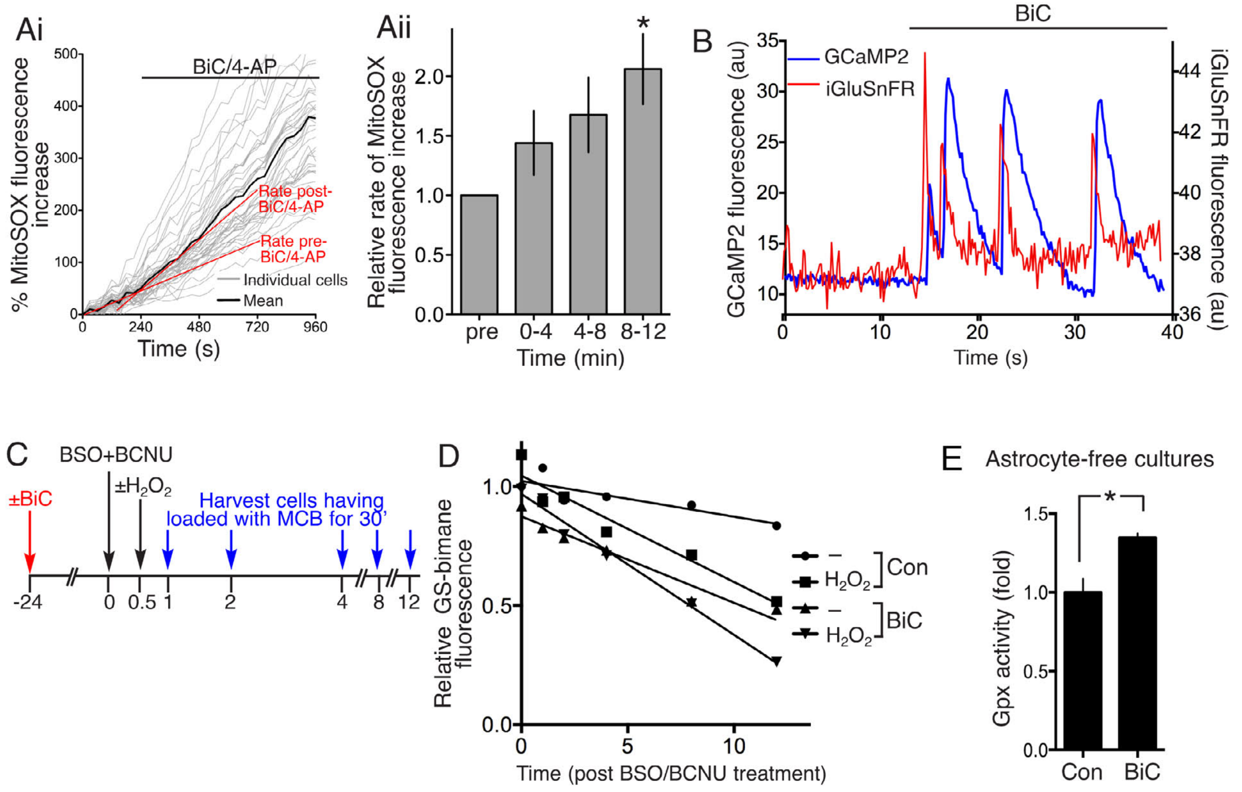

Supplementary Figure 2, relating to Figure 2.

A) Synaptic activity boosts ROS production. Fluorescence images of MitoSOX-loaded neurons taken before/after treatment with bicuculline (50 μM) + 250 μM 4-aminopyridine (BiC/4-AP). Rate of fluorescence increase (relative to initial fluorescence) was calculated before and after BiC/4-AP treatment, over consecutive 4 minute time windows. Left (Ai) shows example trace of a single experiment; right (Aii) shows quantitation of n=3 experiments (*p=0.023). Note that since MitoSOX oxidation, and mitochondrial oxidative stress in general, can arise from ROS generated in the cytoplasm [Mikkelsen, 2003 #3156;Dryanovski, 2013 #3234], MitoSOX oxidation is simply a metric of cellular ROS, and not of mitochondrial ROS in particular. **B)** Cultures of primary astrocytes were transfected with a iGluSnFR vector, after which primary cortical neurons were plated on top. After 7 days, the cultures were transfected with a GCaMP2 vector. In the example shown, a field containing a GCaMP2-expressing neuron an a nearby iGluSnFR-expressing astrocyte were imaged before and after BiC treatment. Note that we confirmed that the astrocyte was indeed expressing iGluSnFR (and not GCaMP2) by co-transfecting mCherry with the GCaMP2. Also we added the glutamate transport inhibitor TBOA (200 μM) and observed a dramatic increase specifically in the iGluSnFR signal size and duration (data not shown). **C)** A schematic outlining the protocol for measuring the rate of decline of GS-bimane signal under the conditions shown in Fig. 2d. **D)** An example of a single experiment performed which contributed to the pooled averaged data shown in Fig. 2c. **E)** Astrocyte-free neuronal cultures were treated ± BiC/4-AP for 24h and a GPX assay performed. *p=0.0107 (n=8)

Fig. S3

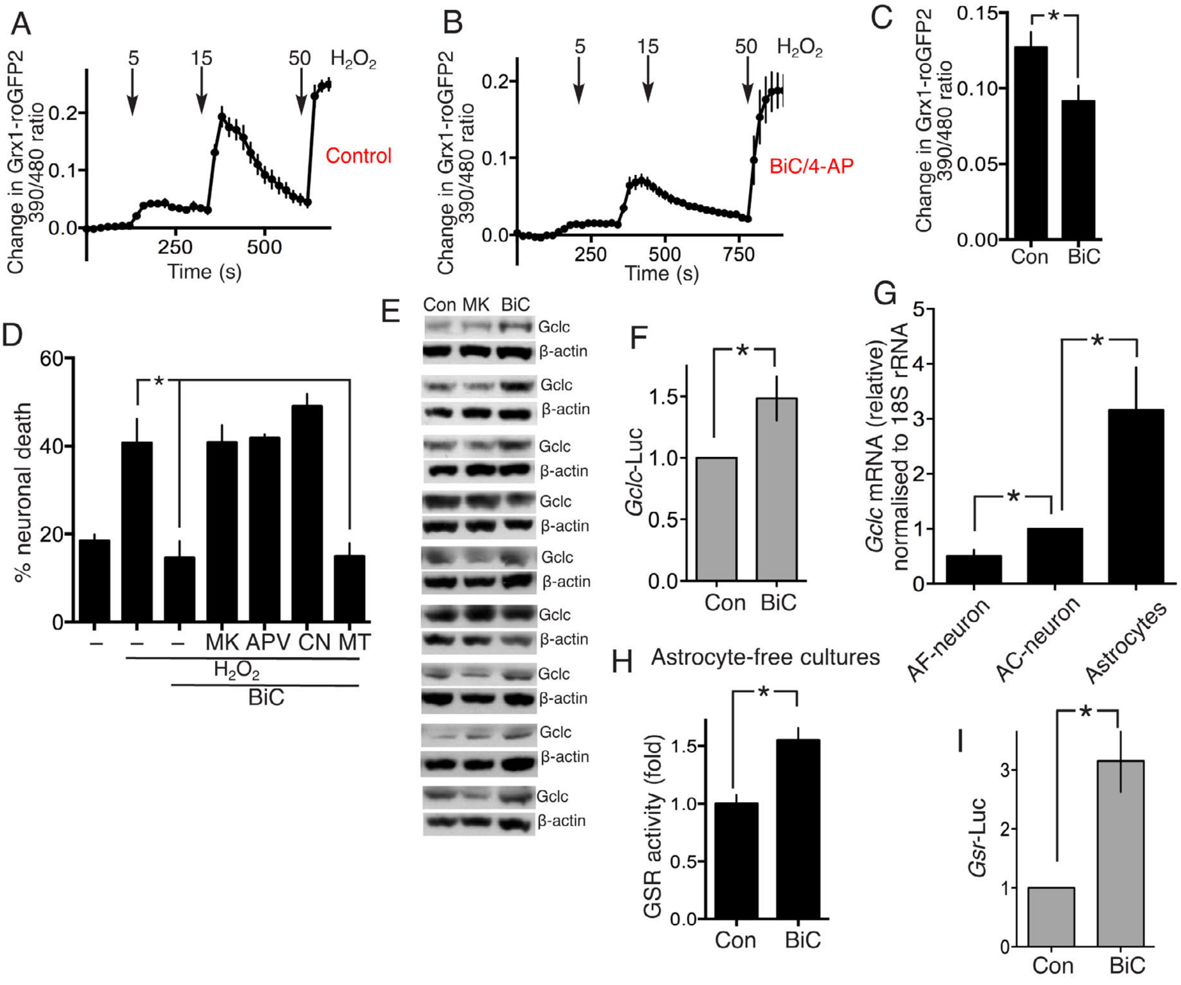

Supplementary Figure 3, relating to Figure 3.

A-C). Neurons expressing Grx1-roGFP [Gutscher, 2008 #3204] treated ± BiC/4-AP for 24h were subject to live cell imaging during treatment with H_2O_2 at the indicated concentrations. Pairs of images were taken (ex 387±5 and 494±10; em 530±10 in both cases) and the ratio calculated. Note that the responses of cells to 5 μM H_2O_2 were very variable and sometimes non-existent, while the responses to 50 μM H_2O_2 were frequently at the limit the dynamic range of the reporter, especially for control cells. Therefore, the response of neurons to 15 μM H_2O_2 was measured (C). *p=0.024 (n=10). **D)** Neurons were treated with ± BiC/4-AP in the presence where indicated of MK-801 (μM), APV (AP, 100 μM), CNQX (CN, 10 μM), MTEP (MT, 1 μM), then treated with 100 μM H_2O_2 with cell death analysed 24 h later. *p<0.0001, <0.0001, 1WA-Fph (n=3). **E)** Western blots relating to Fig. 3I. **F)** Synaptic activity activates the *Gclc* promoter. Cortical neuronal cultures were transfected with firefly a luciferase-based reporter, plus a TK promoter-drive Renilla luciferase-based transfection control. 48 h post-transfection neurons were stimulated with BiC/4-AP and firefly luciferase activity measured, normalised to renilla control *p=0.049, (n=4). **G)** Neurons and astrocytes express different levels of Gclc. Gclc mRNA, normalized to 18s rRNA, was calculated for astrocyte free (AF) cortical neuronal cultures, astrocyte containing (AC, 5-10%) cortical neuronal cultures, and cortical astrocytes, all prepared from the same starting cortical material. For each experimental replicate, expression levels in AF-cultures and astrocytes were normalised to those in mixed AC-cultures. *p=0.0003, 0.049 (n=5-7). **H)** GSR activity in astrocyte-free neuronal cultures treated ± BiC/4-AP. *p=0.0032, (n=5). **I)** Synaptic activity activates the *Gsr* promoter. Experiment performed as per Supplementary Fig. 3f. *p=0.026, (n=4)

Fig. S4

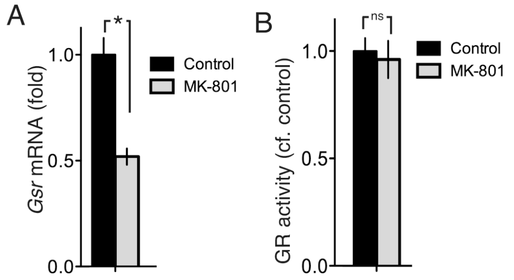

Supplementary Figure 4, relating to Figure 5.
A,B) Blockade of NMDARs decreases Gsr mRNA expression *in vivo* but GR activity is not lowered. P7 rats received two consecutive intraperitoneal injections of saline vehicle or 0.5 mg/kg MK-801 at 0 and 8 h. 12 h after the first injection, rats were sacrificed and frontal cortices were collected and snap-frozen in liquid nitrogen. Gsr mRNA was analysed by qPCR, GR activity as per methods. *p=0.0042, (n=4).
